# Marital status, living arrangements, and mortality in middle and older age in Europe

**DOI:** 10.1007/s00038-020-01371-w

**Published:** 2020-04-29

**Authors:** Pilar Zueras, Roberta Rutigliano, Sergi Trias-Llimós

**Affiliations:** 1 Centre d’Estudis Demogràfics (a Member of the CERCA Programme/Generalitat de Catalunya), Barcelona, Spain; 2 Population Research Centre, Faculty of Spatial Sciences, University of Groningen, Groningen, The Netherlands; 3 Faculty of Epidemiology and Population Health, London School of Hygiene and Tropical Medicine, Keppel Street, London, WC1E 7HT UK

**Keywords:** Mortality differences, Marital status, Partnership status, Living arrangements, Family systems, Welfare states, Europe

## Abstract

**Objectives:**

We study the role of marital status and living arrangements in mortality among a 50+ population living in Europe by gender and welfare states.

**Methods:**

Using data from waves 4, 5, and 6 of the Survey of Health Age and Retirement in Europe (*n *= 54,171), we implemented Cox proportional hazard models by gender and age groups (50–64 and 65–84). We estimated pooled models and separated models for two regions representing different welfare states (South-East and North-West).

**Results:**

Among people aged 50–64, nonpartnered individuals (except never-married women) showed a higher mortality risk as compared with those partnered. Among the older population (65–84), divorce was associated with higher mortality among men, but not among women, and living with someone other than a partner was associated with higher mortality risk as compared to those partnered. In the South-East region living with a partner at ages 50–64 was associated with lower mortality.

**Conclusions:**

Partnership and residential status are complementary for understanding the role of family dimensions in mortality. The presence of a partner is mortality protective, especially among 50–64-year-old men in South-East Europe.

**Electronic supplementary material:**

The online version of this article (10.1007/s00038-020-01371-w) contains supplementary material, which is available to authorized users.

## Introduction

Increasing life expectancy together with family changes (e.g., rising divorce rates, cohabitation, nonmarital fertility, and solo living) is leading to growing diversity in family constellations, marital status, and living arrangements in mid- and later life (Carr and Utz 2020; Esteve et al. 2020). There is an extensive and consistent body of the literature that finds a mortality advantage for married people. However, less is known about the health outcomes associated with both living arrangements and household composition (see Hank and Steinbach 2018 for a review). Although living arrangements and marital status in both mid- and later life largely result from individual choices over the life course, institutional and normative factors also play a role (Pfau-Effinger 2005). However, mainly due to data limitations, the variation in the association between family structure and mortality across different welfare states is currently under researched (Hank and Steinbach 2018; Requena and Reher 2020). This study contributes to the growing body of the literature examining the relationship between family structure, living arrangements, and mortality. The first contribution of the paper is the use of both marital status and living arrangements in a sample of middle-aged and older Europeans. The second contribution is the measurement of the associations between family structure and mortality in two different European welfare states. This study benefits from the Survey of Health, Ageing and Retirement in Europe (SHARE) data (Börsch-Supan et al. 2013), which allowed us to include family, health, and socioeconomic variables in the analysis of mortality in and within Europe by distinguishing two big regions with different welfare states: North-West and South-East.

### Background

Survival at adult and later ages is positively influenced by the quantity and the quality of both social relationships and support (Holt-Lunstad et al. 2010; Shor et al. 2013). Among the extended social net, family members, particularly partners and children, play a crucial role in this association (Giudici et al. 2019; Modig et al. 2017). Differential mortality by marital status has largely been investigated and has shown the benefits of being married. These benefits are greater among men than among women and in younger than in older age groups (e.g., they decrease after the age of 65) (Franke and Kulu 2018; Hank and Steinbach 2018; Hu and Goldman 1990; Murphy et al. 2007; Rendall et al. 2011; Shor et al. 2012; Staehelin et al. 2012). Yet, gender differences between married and nonmarried people at older ages are less evident (Manzoli et al. 2007; Shor et al. 2012). Although research about difference in mortality between never-married, divorced, and widowed individuals is inconclusive, there is a consistent literature that finds elevated mortality risks among divorced people, especially among working age men (Hu and Goldman 1990; Koskinen et al. 2007; Manzoli et al. 2007; Murphy et al. 2007).

There is evidence that the benefits of marital status on health operate through different mechanisms such as health selection, health behaviors, social and economic support, and social control. These mechanisms are found to be stronger among men (Bourassa et al. 2019; Drefahl 2012; Manzoli et al. 2007; Umberson 1987). Previous studies have identified selection, protection, and homogamy effects of being partnered on mortality. At younger ages, there is a positive selection of the wealthier and healthier individuals into marriage (Rendall et al. 2011) and, subsequently, a negative selection out of union for those who are less healthy or have risky behaviors (Umberson 1987). Other studies focused on the protective effect of marriage, which discourages health-damaging behaviors (Franke and Kulu 2018; Guner et al. 2014; Koskinen et al. 2007; Murray 2000). In particular, smoking behavior is associated with marital dissolution, and it partly explains earlier mortality among separated and divorced mature and older people (Bourassa et al. 2019). Thus, the combined effects of selection into and out of marriage and the cumulative advantage of older adults who have been living with a partner for a long time provide possible explanations for different risks of mortality by marital status (Hu and Goldman 1990). Furthermore, similar lifestyles and educational and social homogamy between partners are assumed to equally shape the risk of dying of both partners, consequently explaining the elevated mortality among widowed people (Drefahl 2012; Manzoli et al. 2007; Marmot 2005). Despite the identification of the mechanisms linking marital status and mortality, the benefits of being married on survival persist even after adjusting for socioeconomic factors, objective health, and smoking behavior (Pijoan-Mas and Ríos-Rull 2014).

These findings suggest that other mechanisms—related to union formation—beyond selection, protection, and homogamy, play a role in understanding the survival advantage of marrieds. Given the diversity of partnership situations and family structures, it would be worth considering the role of other family dimensions. Living arrangements, household composition, and the number of children offer a proxy for the type of social relationship, the family resources, and their association with differential mortality across population groups. For example, the household size and its structure (e.g., the presence of dependent children) reduce mortality variation across partnership status only for men (Franke and Kulu 2018). Yet, living alone results in the highest risk of mortality among middle-aged men, upholding the marriage advantage hypothesis (Staehelin et al. 2012). A study for England and Wales found that the health benefits of living with a partner were largest for those aged 50–64 and lowest for those aged 65–85, and the differences in mortality persisted when household size and presence of children were accounted for Franke and Kulu (2018).

It is also known that parents live longer than nonparents and that middle-aged childless women experience higher risks of mortality. Previous studies show a U-shaped relationship between the number of children and mortality risk, and women with either low or moderate number of children show also lower risk of death (Doblhammer 2000). The mortality benefits derived from the number of children were explained by nonexclusive biomedical and social mechanisms (Barclay and Kolk 2019; Doblhammer 2000; Jaffe et al. 2009), including socioeconomic and health selection, risk-avoiding behavior associated with parenthood, and the potential supply of economic and social support at older ages provided by children (Barclay and Kolk 2019; Friedman and Mare 2014; Hank and Steinbach 2018).

Overall, the effect of family support as one of the most beneficial for individuals’ survival appears to be consistent across societies (Holt-Lunstad et al. 2010). However, most research about differential mortality by marital status or living arrangements has a national scope and overlooked a comparative perspective (see Hu and Goldman 1990; Murphy et al. 2007; Noale et al. 2005; Valkonen et al. 2004 for some exceptions). Therefore, understanding the interrelation between partnership or residential status and mortality across societal settings remains an open question (Hank and Steinbach 2018; Requena and Reher 2020).

Specifically, little is known about the associations between family structures and mortality across European regions, as they are characterized by dissimilar family systems and welfare states. These regional contexts result in different levels of intergenerational co-residence, social norms, attitudes toward family support, and public policies (Pfau-Effinger 2005). Regional norms can influence the way in which social relationships, support, and care provision are understood. Following this reasoning, a previous study found positive associations between social vulnerability and mortality in Western and Southern European countries but not in the Nordic ones (Wallace et al. 2015). Furthermore, a meta-analysis found different mortality risk associated with widowhood across European regions (Shor et al. 2012). Overall, the health benefits associated with family structure are found to be stronger in familialistic countries, i.e., South-East Europe, with less generous welfare states and where more people subscribe to norms of strong family obligations, than in more individualistic countries in the North-West, with more socially oriented welfare states (Requena and Reher 2020). Because of these well-established differences in the role of family and welfare states across European regions, studying the role of both marital status and living arrangements in health outcomes is assumed to be particularly relevant.

### Aims

This study examines the relationship between family structure, i.e., marital status, living arrangements, and number of children, and mortality risk for middle-aged and older European population by age group and gender. The first objective of this research is to account for the identified selective, protective, and homogamy effects described above in the associations between family structures and mortality. The second objective of this research is to study, for the first time, these associations from a comparative perspective exploring potential differences within main welfare states in Europe.

## Methods

This study analyzed data from waves 4, 5, and 6 of the Survey of Health Age and Retirement in Europe (SHARE), collected in 2011, 2013, and 2015. SHARE is a biennial longitudinal individual-level data of nationally representative samples of population aged 50 or older across Europe, collected from 2004 onward (Börsch-Supan et al. 2013). It includes information about a wide range of socioeconomic, demographic, and health topics, as well as information of deceased respondents from end-of-life interviews.

We selected individuals who were 50–84 years old when they were first observed and whose survival status in the subsequent wave was known (76.4% between wave 4 and 5 and 80.0% between 5 and 6). We grouped our sample in two age groups: 50–64 and 65–84 (age at the beginning of the risk window). We excluded 1 221 individuals (2.20%) with missing information on our main explanatory variables (see details below). We ended up with a final sample of 29 917 women and 24 254 men.

### Variables

The process time in our model was the age of individuals expressed in months, and the outcome variable was time to death. With this approach, we considered the effect of age on mortality while not specifying any functional form for the hazard function (see Thiébaut and Bénichou 2004 for further detail). Marital status (married or partnered, divorced, never-married, and widowed) and living arrangements (living with a partner in the household (either with or without others), living alone, and living with someone other than a partner) were defined at the beginning of each person-month of the exposure to death. We included as covariates education and two time-varying characteristics: smoking behavior and self-rated health (SRH).

The control variables were coded as follows. Educational attainment: primary (ISCED 0–2), secondary (ISCED 3–4), or tertiary (ISCED 5–6). Smoking was coded as a binary variable. SRH was recoded into three categories: fair and poor, good, and very good and excellent. We also included the number of children distinguishing between childless, having one child, and having two or more children. Finally, we created a control variable that grouped all countries into four regions: North (Sweden, Netherlands, Denmark), West (Austria, Germany, France, Switzerland, Belgium, and Luxembourg), South (Spain, Italy, and Israel), and East (Czech Republic, Slovenia, and Estonia), which was used to adjust the first models. We, then, gathered those into two big regions: North-West and South-East to run the final models in parallel for different family and welfare contexts.

### Statistical methods

We performed separate Cox proportional hazard models for both genders and age groups (50–64 and 65–84) to estimate the hazard ratios (HR) of mortality by marital status and living arrangements. We adjusted for the clustered structure of the data by computing robust standard errors (Cleves et al. 2008). In a first set of models, we focused on marital status and we ran nested specifications to adjust by both sociodemographic characteristics and health variables (1). Formally, for each individual *i* of age *t* (measured in months), the hazard of dying was modeled as follows:

1 $$h_{i} \left( t \right) = h_{0} \left( t \right)*{ \exp }\left( {x_{i,1} \beta_{i,1} + x_{i,2} \beta_{i,2} + x_{i,3} \beta_{i,3} + x_{i,4} \beta_{i,4} + x_{i,5} \beta_{i,5} } \right)$$

where $h_{i} \left( t \right)$ was the hazard for individual *i* at age *t;*$X_{i}$ is a vector of covariates with coefficients $\beta$. Specifically, starting from the null model (only marital status,$x_{i,1}$, we added educational level ($x_{i,2}-{\text{M}}2$), geographical region ($x_{i,3}-{\text{M}}3$), SRH and smoking behavior ($x_{i,4}-{\text{M}}4$), and the number of children ($x_{i,5}-{\text{M}}5$). Finally, $h_{0} \left( t \right)$ was the baseline hazard, i.e., the hazard when the vector $X_{i} = 0$.

In the second set of models, we focused on living arrangements and followed the same nested specifications [see formula (1)]. We examined the relationship between living arrangements and mortality both in the pooled model, including all individuals from all countries, and in separate models for different welfare states.

## Results

### Descriptive statistics

Table 1 shows descriptive statistics by age, sex, and region, stratified for our main explanatory variables (marital status and living arrangements).

**Table 1:** Descriptive statistics for the dependent variables. Survey of Health, Ageing and Retirement in Europe, waves 4 (2011), 5 (2013), and 6 (2015) for Sweden, Netherlands, Denmark, Austria, Germany, France, Switzerland, Belgium, Luxembourg, Spain, Italy, Czech Republic, Slovenia, and Estonia

	Women						Men					
	50–64			65–84			50–64			65–84		
	Person-months	*n*.	*N.* deaths	Person-months	*n*.	*N.* deaths	Person-months	*n*.	*N.* deaths	Person-months	*n*.	*N.* deaths
**South-East**												
Living arrangements												
Partner	158,105	5259	74	112,313	3929	218	12,937	4263	147	137,300	4774	605
Alone	28,802	868	25	69,402	2230	205	14,814	479	41	18,815	617	92
Other than partner	18,676	562	12	23,468	734	100	7641	251	19	5214	171	37
Marital status												
Partnership	154,400	5140	74	113,752	3962	231	124,054	4110	138	135,600	4728	598
Divorced	23,316	711	15	13,635	439	26	12,937	409	22	6050	202	31
Single	9979	321	5	7763	262	24	11,786	390	33	5833	206	27
Widow	17,888	517	17	70,033	2230	242	2996	84	14	13,846	426	78
**Total sample**	205,583	6689	111	205,183	6893	523	151,773	4993	207	161,329	5562	734
**North-West**												
Living arrangements												
Partner	188,459	6845	61	116,763	4330	131	160,673	5813	75	145,864	5406	317
Alone	45,858	1585	21	80,628	2733	141	30,957	1071	18	31,566	1099	95
Other than partner	16,895	592	4	8100	250	21	6684	228	2	2541	82	11
Marital status												
Partnership	180,683	6576	57	119,249	4381	145	152,166	5507	73	144,582	5346	320
Divorced	36,002	1262	16	19,579	689	20	22,692	786	11	11,711	419	32
Single	17,619	622	6	8736	306	17	18,808	666	6	7775	279	16
Widow	16,908	562	7	57,927	1937	111	4648	153	5	15,903	543	55
**Total sample**	251,212	9022	86	205,491	7313	293	198,314	7112	95	179,971	6587	423

For each region-, age-, sex-specific combination, we counted at least 86 deaths (North-West, 50–64, female) and 151,773 person-months at risk (over 12,640 person-years).

**Fig. 1:**
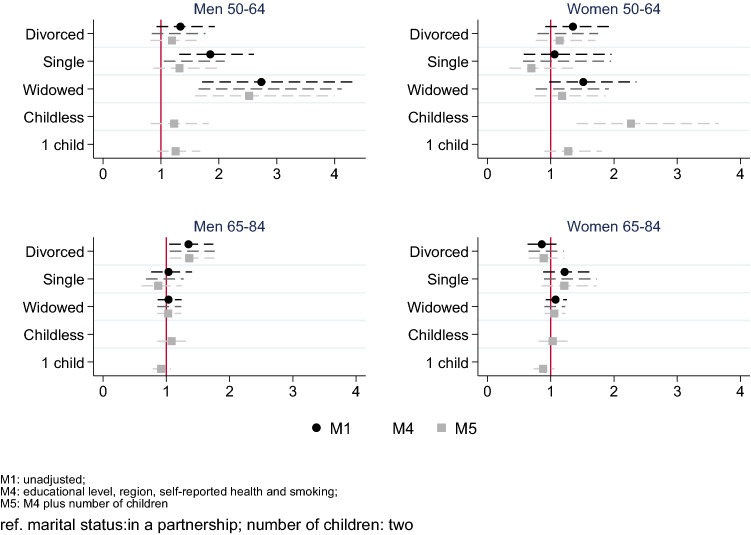
Hazards ratio (HR) of mortality by marital status and by number of children. Survey of Health, Ageing and Retirement in Europe, waves 4 (2011), 5 (2013), and 6 (2015) for Sweden, Netherlands, Denmark, Austria, Germany, France, Switzerland, Belgium, Luxembourg, Spain, Italy, Czech Republic, Slovenia, and Estonia

Figure 1 shows the associations between marital status and number of children, and mortality. Among individuals aged 50–64, all nonmarried categories (except never-married women) were associated with higher mortality risk as compared with marrieds. These estimates were statistically significant among never-married and widowed men (HR 1.85, 95% CI 1.32–2.60 and 2.73, 1.71–4.37, respectively). Adding SRH reduced the HR, especially for never-married men (which remained significant) and for divorced and widowed women. In the fully adjusted model, which controlled for the number of children, only excess mortality of widowers remained high (HR 2.52, 1.59–3.99). Being childless was associated with higher mortality risk among younger women as compared to those having two or more children (HR 2.27, 1.41–3.66).

Among those aged 65–84, divorce was associated with higher mortality among men (M1 HR 1.35, 1.05–1.74), but not among women (M1 HR 0.86, 0.63–1.17), whereas being never-married was positively associated with mortality among women (M1 HR 1.22, 0.88–1.69). These results remained steady after adding the controls. The number of children was not associated with distinct mortality risk.

### Associations between living arrangements and mortality

**Fig. 2:**
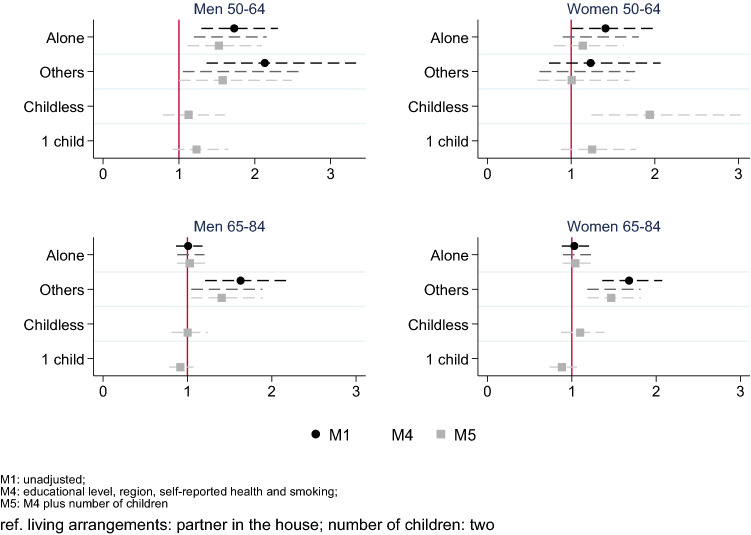
Hazards ratio (HR) of mortality by living arrangements and by number of children. Survey of Health, Ageing and Retirement in Europe, waves 4 (2011), 5 (2013), and 6 (2015) for Sweden, Netherlands, Denmark, Austria, Germany, France, Switzerland, Belgium, Luxembourg, Spain, Italy, Czech Republic, Slovenia, and Estonia

Figure 2 shows the associations between living arrangements and number of children, and mortality. For both men and women aged 50–64, those living alone showed excess mortality (M1 HR 1.73, 1.30–2.30, and 1.41, 1.01–1.97, respectively) that reduced after adjusting for the control variables, while remaining significant for men (HR 1.53, 1.11–2.09), but not for women (HR 1.14, 0.80–1.63). Men living with others without a partner have higher mortality risk as compared to those living with a partner (M1 HR 2.13, 1.36–3.34) and differences diminished after adjusting for SRH (M4 HR 1.65, 1.05–2.58).

Among the older population (65–84) living with others without a partner was associated with higher mortality risk compared to living with a partner. HR decreased after adjusting for education, SRH, and smoking among men and for region and SRH among women, but slightly changed when controlled by the number of children (M5 HR 1.41, 1.04–1.89 among men and 1.47, 1.18–1.82 among women).

### Associations between living arrangements and mortality by region

**Fig. 3:**
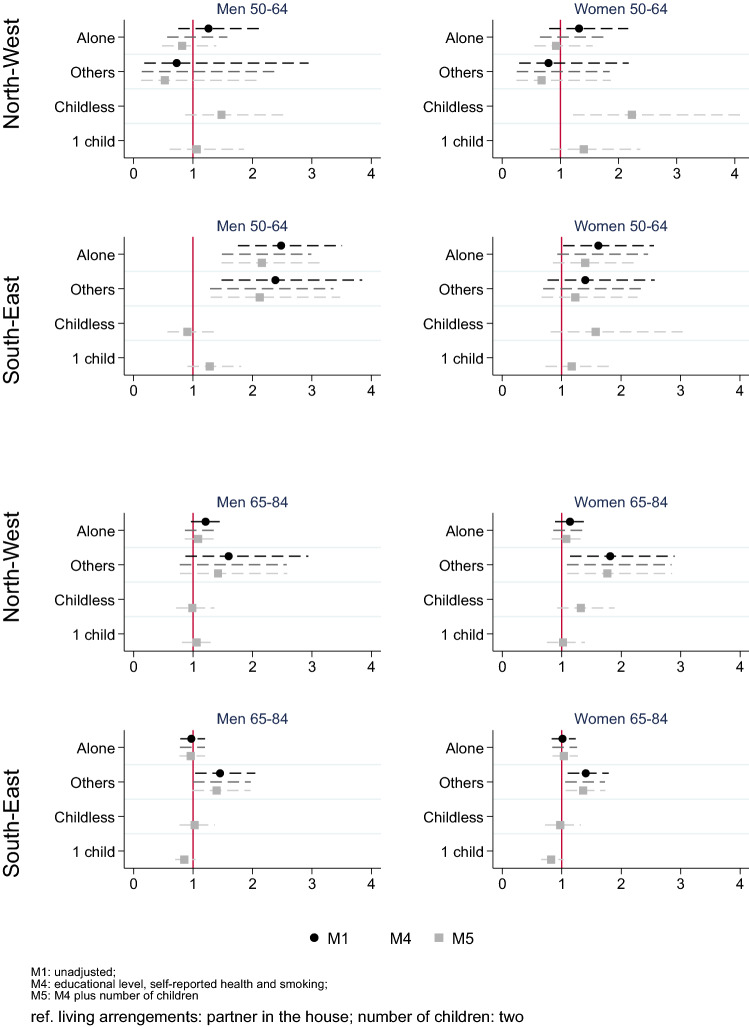
Hazards ratio (HR) of mortality by living arrangements and number of children by regions. Survey of Health, Ageing and Retirement in Europe, waves 4 (2011), 5 (2013), and 6 (2015) for Sweden, Netherlands, Denmark, Austria, Germany, France, Switzerland, Belgium, Luxembourg, Spain, Italy, Czech Republic, Slovenia, and Estonia

Figure 3 shows the region-specific associations between living arrangements and number of children, and mortality. Among population aged 50–64 living in North-West countries, there were no benefits of living with a partner (Fig. 3). However, in the South-East region living with a partner was found to be protective, especially among men in all model specifications (M5, HR for living alone 2.04, 1.40–2.97 and with others 2.10, 1.30–3.40). Childless women had higher mortality risk in both regions (North-West region HR 2.17, 1.19–3.98; South-East region HR 1.44, 0.74–2.78).

Among the older population, we found higher mortality risk among those who were living with others without a partner as compared to those living with a partner. After adjusting for the covariates these HRs remained similar and significant only among women (M5, HR 1.64, 1.02–2.63 in the North-West and HR 1.34, 1.06–1.71 in the South-East). Finally, the results suggest a protective effect of having an only child in South-East region (HR 0.87, 0.72–1.06 among men and HR 0.81, 0.65–1.02 among women).

Overall, the direction of the association between both the socioeconomic and the health control variables, and mortality was in line with our expectations: a mild negative association with education, a strong negative association with SRH, and higher mortality of smokers as compared to nonsmokers.

## Discussion

This study examined how marital status and living arrangements are related to (1) differences in mortality in a sample of European adults aged 50 and over and (2) potential variation across European welfare states. The results show the importance of being partnered, especially among middle-aged men. At older ages, living with someone other than the partner is associated with higher mortality as compared to living with the partner for both genders. Results for the two European welfare regions examined suggest living with a partner to be mortality protective in the South-East region and especially among middle-aged men, but not in the North-West. Furthermore, older people living with others than the partner show excess mortality in both regions.

Our results on the excess mortality among middle-aged nonmarried individuals but not among older individuals are consistent with previous findings suggesting wider differences among men than among women and among the working aged population than among the over 65 (Franke and Kulu 2018; Guner et al. 2014; Hu and Goldman 1990; Koskinen et al. 2007; Manzoli et al. 2007; Murphy et al. 2007; Murray 2000; Rendall et al. 2011). In line with earlier research (Pijoan-Mas and Ríos-Rull 2014), differential mortality by marital status persisted even after adjusting for socioeconomic, health status, and smoking behavior variables. This finding upholds the protection and selection mechanisms related to marriage mortality advantage. Indeed, at ages 50–64, both widowed men and women, and never-married men experienced higher mortality risk, which generally diminished after adjusting for confounders, especially SRH, except for widowed men (HR 2.73). Probably young widowers are a highly selected group of more disadvantaged men in terms of health and SES who have not re-partnered despite their young age. In addition to the protective effect of both marriage and socioeconomic homogamy, the sharing living conditions of couples (e.g., healthcare access, health literacy) could explain the higher mortality risk among widowed men.

Among the oldest population, we found higher mortality risk for divorced men, but not for women, which contrasts with earlier findings (Koskinen et al. 2007; Manzoli et al. 2007; Murphy et al. 2007). Our results are consistent with the literature finding that there are more pronounced differences in lifestyles between married and divorced men, who are more likely to engage in unhealthy and risky behaviors, as compared to these differences between divorced and married women (Bourassa et al. 2019; Umberson 1987). However, adjusting for SRH, smoking behavior, and education did not reduce the HR for older divorced men (HR 1.35). A possible explanation for this gender difference among the oldest cohorts could be a diverging selection by income. That is, men who divorced and did not partner again might be less wealthy (negative selection), whereas women who divorced and stayed nonpartnered might be wealthier and could afford to live by themselves (positive selection) (Shafer and James 2013).

In line with previous results, men aged 50–64 showed the strongest benefits of living with a partner as compared to living alone or with someone other than a partner (Franke and Kulu 2018; Staehelin et al. 2012), while for women only living alone was associated with higher mortality risk in the crude models but not in the adjusted models. Results show that the presence of a partner is more critical for middle-aged men than for women, consistently with previous research (Koskinen et al. 2007; Staehelin et al. 2012) that suggest a central role of wives in men’s social support and network connection.

At older ages, those living alone did not have different mortality risk as compared to those living with a partner, especially in South-East Europe. In the North-West region the elevated mortality risk of those living alone reduces after adjusting for health status. This suggests that there is a positive selection of those living alone at older age, and that the level of independence and good health required to live alone may be higher in South-East than in North-West welfare states (Requena and Reher 2020). These results should be taken cautiously because recording mortality in survey data seems harder among those people living alone (Chatfield et al. 2005). However, this seems not to be the case in our sample (see Supplementary Table 1).

Furthermore, individuals living with someone other than a partner presented higher mortality risk (except for middle-aged women) compared to those living with a partner. Adjusting for controls, especially for SRH, reduced those differences, which suggests that old people in those living arrangements might have poorer health and functional conditions (Requena and Reher 2020). In other words, individuals living with someone other than a partner might be less capable of living alone. This latter finding holds also in the South-East, but not in the North-West region (except for older women), and could be related to social norms and distinct regional patterns of co-residence with adult children and institutionalization (as discussed in Requena and Reher 2020). Dependent individuals tend to live more at home (with relatives or caregivers) in Southern and Eastern Europe, whereas these individuals are more likely to enter residential care in Western and Nordic systems (EUROSTAT; Laferrère et al. 2013). Future research should further investigate whether differential associations between the family structure and mortality exist across European welfare states.

Finally, we expected to find evidence of social support related to the number of children among the older population (65–84). Having a child suggested a protective effect, consistent with higher involvement of only children in parental care (Campbell and Martin-Matthews 2003), but only in the familialistic welfare state. Previous studies did not find strong evidence on the importance of social support in explaining higher longevity of parents in Sweden (Barclay and Kolk 2019; Modig et al. 2017). However, gender similarities of elevated mortality risk among the childless middle-aged population in the North-West suggest that protective social mechanisms are in action (e.g., healthier lifestyles of parents compared to nonparents), whereas cross-national higher mortality of childless middle-aged women can be explained by biomedical factors (Barclay and Kolk 2019; Doblhammer 2000; Jaffe et al. 2009).

This study took advantage of the longitudinal aspect of SHARE data to study the associations between marital status, living arrangements, and differential mortality across different European contexts. As compared to vital statistics registers or linked censuses data, SHARE data provide information on marital status and other family resources, together with social, socioeconomic, and health measures. As for every longitudinal analysis attrition might represent an important limitation. In our case, because of the relatively short follow-up period (around 2 years) we could follow > 75% of the cases. Furthermore, those individuals who drop-off of the sample do not report worse health status (see Supplementary Table 1). As many other health surveys, SHARE sample is selected. However, the share of institutionalized individuals is rather low at the age groups of our interest (50–84); i.e., below 8% at age 80–84 in the countries under study (EUROSTAT). Nonetheless, following a previous study (Solé-Auró et al. 2015) we compared SHARE mortality data with mortality register data. This comparison resulted in SHARE mortality to be slightly lower than population-level mortality (Supplementary Fig. 1). Finally, to make sure that our sample selection does not bias our results, we run some sensitivity analysis. Specifically, we checked the distribution of the missing values over the sample and we re-run the models including a specific category for missing values for each variable. Our results prove robust and the distribution of the missing values does not highlight any selection bias.

### Conclusion

The relationship between family structure and mortality in different welfare states has not been studied before. Overall, our results confirm that, regardless of welfare states, being partnered is associated with lower mortality, especially among middle-aged men in South-East Europe. Moreover, this advantage persisted after accounting for smoking behavior and socioeconomic and health status, which are related to well-known selection and protection mechanisms of being partnered. At older ages, living arrangements are more strongly associated with mortality than marital status, especially among men. This suggests that individuals who are not living alone nor with the partner show higher mortality risk as compared to those who live with their partner. Furthermore, our results pointed at living arrangements and the number of children as important factors for mortality risk in South-East Europe, where the welfare system is less generous than in North-West Europe and in which the well-being of older individuals depends more on their family members. In sum, both marital status and living arrangements are complementary variables in the complex associations between family and mortality at middle and old age in Europe.

## Electronic supplementary material

Below is the link to the electronic supplementary material.

Supplementary material 1 (DOCX 105 kb)
