# Supplementary material for: Marital status, living arrangements, and mortality in middle and older age in Europe
Source: Int J Public Health. 2020 Apr 29;65(5):627–36. doi: 10.1007/s00038-020-01371-w (PMC7360666; doi:10.1007/s00038-020-01371-w)
Supplement: Supplementary file 1 — Supplementary material 1 (DOCX 105 kb) [file 38_2020_1371_MOESM1_ESM.docx]

**Supplementary material**

**Supplementary Table 1**. Comparison of prevalence of several variables in the followed-up and the lost to follow-up samples (with 95% CI), Survey of Health, Ageing and Retirement in Europe (SHARE), waves 4-5 and 5-6.

| Age group 50-64 |  |  |  |  |
| --- | --- | --- | --- | --- |
|  | Men | | Women | |
|  | Followed up | Lost | Followed up | Lost |
| married | 77.9% (77.3-78.5) | 78.4% (77.3-79.5) | 72.6% (72.0-73.1) | 73.7% (72.6-74.8) |
| widowed | 2.0% (1.8-2.2) | 1.9% (1.5-2.2) | 7.2% (6.8-7.5) | 6.4% (5.8-7.0) |
| divorced | 10.1% (9.7-10.6) | 10.2% (9.4-11.0) | 13.0% (12.5-13.4) | 11.9% (11.1-12.8) |
| never married | 8.9% (8.5-9.3) | 8.0% (7.3-8.7) | 6.1% (5.8-6.5) | 6.4% (5.8-7.0) |
| living alone | 13.2% (12.7-13.7) | 12.3% (11.4-13.2) | 16.2% (15.7-16.7) | 14.9% (14.0-15.8) |
| smokers | 28.7% (28.0-29.4) | 30.0% (28.7-31.2) | 22.3% (21.8-22.9) | 22.5% (21.5-23.6) |
| obesity | 21.8% (21.2-22.4) | 21,0% (19.9-22.1) | 21.1% (20.6-21.7) | 17.9% (17.0-18.9) |
| good self-reported health | 70.3% (69.6-71.0) | 71.0% (69.8-72.2) | 69.9% (69.3-70.5) | 70.0% (68.8-71.1) |
| 2+ chronic | 36.3% (35.6-37.0) | 33.5% (32.3-34.8) | 37.0% (36.4-37.7) | 33.4% (32.2-34.6) |
| Cognitive (good or excellent) | 71.5% (70.9-72.2) | 70.0% (68.8-71.2) | 72.1% (71.5-72.7) | 69.5% (68.3-70.6) |
| Global Activity Limitation Indicator | 36.3% (35.6-37.0) | 34.0% (32.8-35.3) | 39.2% (38.6-39.8) | 37.1% (35.9-38.3) |
|  |  |  |  |  |
| Age group 65-84 |  |  |  |  |
|  | Men | | Women | |
|  | Followed up | Lost | Followed up | Lost |
| married | 80.8% (80.3-81.4) | 81.7% (80.6-82.8) | 55.7% (55.1-56.4) | 58.5% (57.1-59.9) |
| widowed | 8.3% (7.9-8.7) | 7.3% (6.5-8.1) | 31.1% (30.4-31.7) | 28.6% (27.4-29.9) |
| divorced | 5.1% (4.8-5.4) | 5.0% (4.4-5.7) | 8.1% (7.7-8.5) | 6.5% (5.9-7.2) |
| never married | 4.2% (3.9-4.5) | 4.2% (3.6-4.8) | 4.1% (3.8-4.3) | 4.6% (4.0-5.2) |
| living alone | 14.7% (14.2-15.2) | 13.7% (12.7-14.8) | 36.9% (36.2-37.5) | 34.0% (32.6-35.3) |
| smokers | 15.2% (14.7-15.7) | 14.8% (13.8-15.8) | 8.8% (8.5-9.2) | 8.7% (7.9-9.5) |
| obesity | 19.6% (19.1-20.2) | 18.1% (17.0-19.2) | 22.4% (21.8-22.9) | 20.7% (19.6-21.8) |
| good self-reported health | 57.7% (57.0-58.4) | 53.7% (52.3-55.2) | 50.1% (49.5-50.8) | 47.2% (45.8-48.6) |
| 2+ chronic | 54.5% (53.8-55.2) | 54.9% (53.4-56.3) | 62.5% (61.9-63.2) | 60.2% (58.8-61.6) |
| Cognitive (good or excellent) | 75.1% (74.5-75.7) | 74.0% (72.7-75.3) | 76.2% (75.6-76.7) | 74.9% (73.6-76.1) |
| Global Activity Limitation Indicator | 49.0% (48.3-49.7) | 50.4% (48.9-51.9) | 56.6% (56.0-57.3) | 57.8% (56.4-59.2) |

In grey the samples with statistically higher prevalence

**Supplementary** **Figure 1**. Age- and sex-specific probabilities of dying. Comparison between the mean of data from the Human Mortality Database (HMD, for 13 countries included) in 2011 and from the Survey of Health, Ageing and Retirement in Europe (SHARE, 2011-2015)


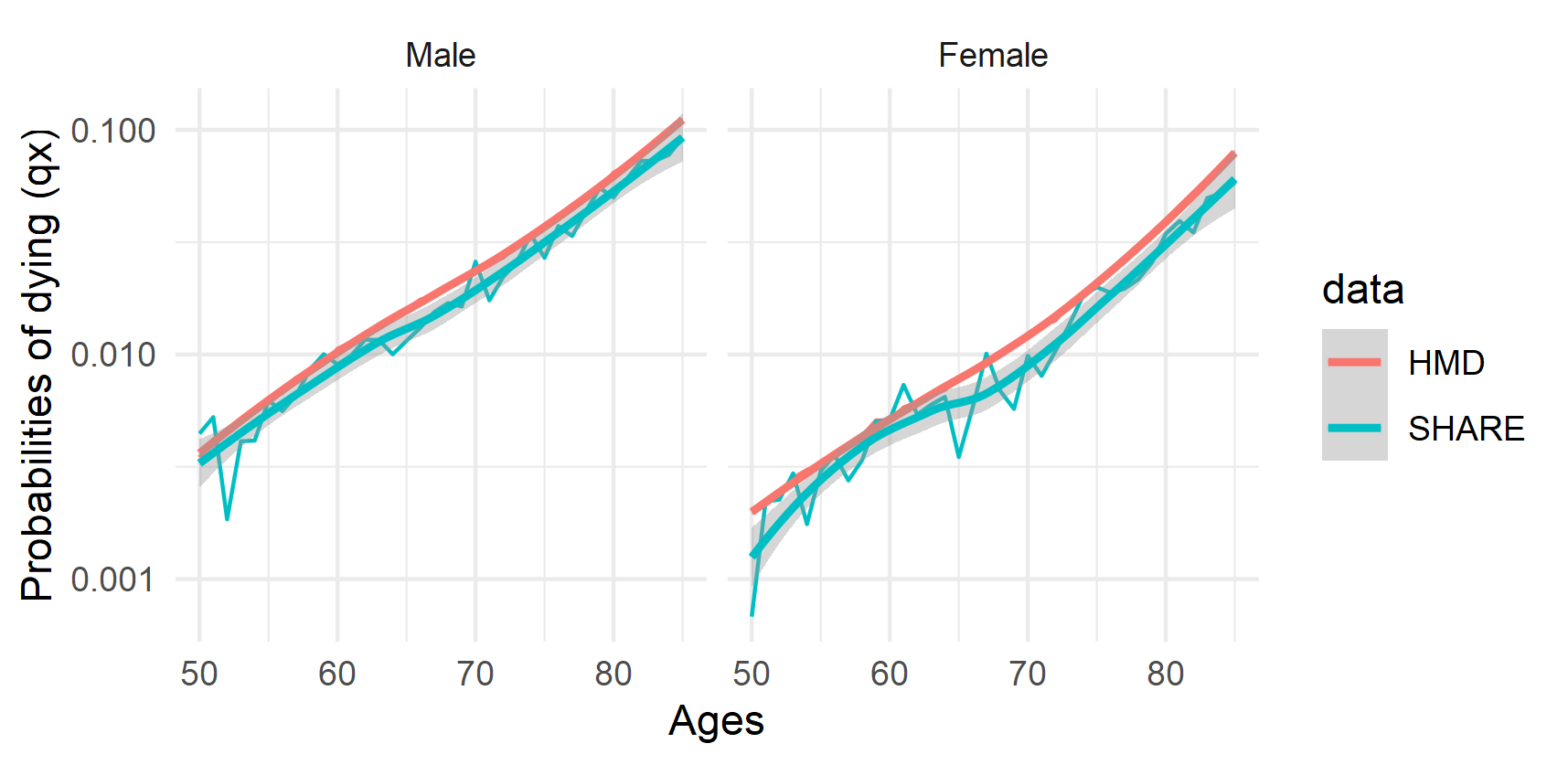


Note: Human Mortality Database (HMD) data for 2011 for the 13 selected countries: Austria, Belgium, Czech Republic, Germany, Denmark, Spain, France, Italy, the Netherlands, Sweden, Switzerland, Estonia and Slovenia.
